# Ethics of brain-computer interface in mental healthcare

**DOI:** 10.3389/fnhum.2026.1846283

**Published:** 2026-08-11

**Authors:** Ajay Chandra, Arkalgud Ramaprasad, Madhavi Rangaswamy

**Affiliations:** 1 School of Psychological Sciences, Christ (Deemed to be University), Bengaluru, India; 2 School of Arts, Humanities, and Social Sciences, Chanakya University, Bengaluru, India; 3 Department of Information and Decision Sciences, University of Illinois, Chicago, IL, United States

**Keywords:** brain computer interface, ethics, framework, mental healthcare, efficiency, effectiveness

## Abstract

Invasive, semi-invasive, and non-invasive brain-computer interfaces (BCI) are playing an increasingly important role in mental healthcare. They play a central role in all the stages of neuro-behavioural interventions, both by the care providers and the caretakers of a patient. The novelty of the technology and its rapid development create complex ethical challenges that must be balanced against the potential gains in efficiency and effectiveness of using the technology in mental healthcare. The paper presents an Ontology of Ethics of Brain-Computer Interface in Mental Healthcare, as a roadmap to guide to balance the ethics, effectiveness, and efficiency of the use of the technology. Like a ‘Google Map’ the ontology presents all the pathways by which the three issues can affect neuro-behavioural interventions using BCI technologies by the variety of agents that deliver mental healthcare. Using the ontology one can determine (a) the desired ethical pathways and reinforce them, (b) the undesired/unethical pathways and redirect them, and (c) discover novel ethical pathways and explore them.

## Introduction

Brain-computer interface (BCI) is emerging as a transformative, closed-loop technology in mental healthcare (Chai et al., 2024; Zhang J. et al., 2024) wherein such care is largely framed within a brain-based disease model. This framing directly motivates the development of closed-loop BCIs and circuit-based neuromodulation for depression and other neuropsychiatric disorders to decode symptom states from neural biomarkers and correct dysfunctional circuits (Oganesian and Shanechi, 2024; Zhang J. et al., 2024). Critiques in clinical psychology and the philosophy of psychiatry argue that biological reductionism has dominated mental healthcare since the 1980s and has narrowed research, policy, and services to the brain alone (Jerotic and Aftab, 2021; Fried, 2022). Empirical work on BCIs highlights that there is little attention to self-experience, self-image, agency, identity, and what it feels like to act through a BCI (Kögel et al., 2019). Human-computer interaction and digital mental health research further emphasize that the technological solutions must be co-designed with the users and clinicians, embedded in therapeutic alliances and service contexts, rather than being treated as stand-alone circuit fixes (Balcombe and De Leo, 2022).

BCIs integrate neural information with appropriate computer science technologies to address mental health conditions, detect abnormal thoughts, provide neuromodulation, conduct therapeutic interventions and treat the patient (Chai et al., 2024; Sellers et al., 2024; Zhang J. et al., 2024; Xiong, 2025). BCIs are increasingly used for diagnosis, neuromodulation, neurofeedback, and assistive communication in mental health and psychiatric care (Karikari and Koshechkin, 2023; Song, 2025). BCI technologies have been effective enablers of direct communication between the brain and an external device. An effective BCI technology reads brain signals, interprets them, and uses them to control a machine/computer, or a prosthetic limb.

The need for BCIs to shift from a brain-level to a multi-level modelling and intervention strategies is gaining ground. A parallel shift is occurring alongside circuit-centric research, with a growing trend to view mental healthcare as an intricate system involving biological, social, and psychological elements, applying methodologies from network and systems science (Fried, 2022). The need for scientific pluralism has pushed the domain towards more system-based, ethically grounded, person-centred, and socially responsible models of mental healthcare and BCI designs.

Given the intricate relationship with the cognitive, conative, and affective processes, the clinical use of BCIs raises distinct ethical, legal, and social considerations that require specific governance. In this context, UNESCO’s recommendations towards the adoption of a global normative framework on the ethics of neurotechnology are a landmark step in governing technologies that directly interface with the human brain and nervous system (UNESCO, 2025). Emerging neurotechnology-specific human rights in terms of mental privacy over neural data, mental integrity against unwanted alteration of mental states, and personal identity against manipulation of agency, and enable cognitive freedom, especially to protect the inner mental domain of individuals, are all critical to enhance mental healthcare and human well-being (Ienca et al., 2022; Ruiz et al., 2024).

BCI technologies have evolved into invasive, semi-invasive, and non-invasive modalities (Zhang H. et al., 2024; Khorev et al., 2024), based on their level of invasiveness and intervention. For example, ECoG, an invasive cortical BCI, where electrodes are placed on the cortical surface to capture high resolution signals have been effective for motor rehabilitation, seizure control and psychiatric conditions (Hramov et al., 2021; Karikari and Koshechkin, 2023; Zhang H. et al., 2024). Similarly, non-invasive EEG assistive and rehabilitation BCIs, like communication devices, cognitive training and robotic control are used for patients with neurodevelopmental disorders, severe disability and conditions with cognitive decline (Papanastasiou et al., 2020; Khorev et al., 2024; Magalhães et al., 2025). The non-invasive systems like EEG and fNRIS headsets operate at a cortical level either using passive or active paradigms to monitor and modulate psychophysiological state (Hramov et al., 2021; Khorev et al., 2024).

BCIs technologies have opened new frontiers in mental healthcare by transforming brain signals into actionable insights for personalised, real-time support. EEG based systems enable reading cortical activity through the scalp to provide neurofeedback in depression, anxiety, affective disorders, attention training, and emotional regulation (Hramov et al., 2021; Angulo Medina et al., 2024; Khorev et al., 2024; Magalhães et al., 2025). BCI’s effectiveness in terms of signal acquisition, feature extraction (e.g., motor imagery), classification and translation, and control over output has been instrumental in significant advances in neurorehabilitation. EEG BCI neurofeedback has been applied to affective disorders and broader mental health rehabilitation, with meta-review data showing promising but heterogeneous outcomes and emphasizing the need for more standardized protocols and outcome metrics (Angulo Medina et al., 2024; Khorev et al., 2024).

Studies on the clinical efficacy and effect size of BCI-based neurorehabilitation have reported their significant role in diagnosis, promotion of neurological recovery, neuroplasticity, improved motor functions, rehabilitation, training, and evaluation of functional status (Chai et al., 2024; Khorev et al., 2024; Ma et al., 2025; Liu Q. et al., 2025). The limited understanding about neural systems, optimal interface mechanisms, and largely mental health BCIs, however makes them especially susceptible to unique ethical challenges.

Ideally, BCI research in mental healthcare should traverse across different systemic levels, have a closed-loop, and integrate Artificial Intelligence (AI) systems. For instance, closed-loop neuromodulation tailored to individual biomarkers for depression, Obsessive Compulsive Disorder (OCD), addiction and other psychiatric disorders, framed as “digital prescriptions” that specify targets, coding/decoding protocols, and interaction paradigms (Chai et al., 2024; Zhang J. et al., 2024). Emerging BCI based digital prescriptions must not only be tailored to each patient’s specific functional impairments (Ma et al., 2025), but also emphasize on factors like the paradigm used (P300 or steady-state visually evoked potentials (SSVEPs) in speech disorders), combination of the interface mechanisms (e.g., TMS or DBS or tDCS) to be used for modulation of emotional dysfunction (Chai et al., 2024). The efficiency and effectiveness of BCI interventions must be weighed against their ethics. In future, the evaluation of ethics, efficiency, and effectiveness will become central to arriving at a scientific plural, integrated and systems-based approach towards neuro-behavioural modulation in mental healthcare.

The 3 Es in terms of Ethics, Efficiency, and Effectiveness of BCIs must be dealt and comprehended in detail. Ethical considerations of BCIs, especially in terms of autonomy, identity, and privacy have been widely articulated. Concerns over ethical standards for invasive and non-invasive BCIs must be differentiated and integrated to develop a unified code of ethics (Zhang H. et al., 2024). For instance, a few patients who are locked-in or unable to communicate may not choose to use BCI technologies, despite its significant benefits (Burwell et al., 2017; Ma et al., 2025).

The effectiveness of BCIs in terms of control, communication, rehabilitation, recovery and enhancement functions have often been evaluated and have raised concerns. Adverse effects of BCI’s on mental and physical health of users should be systematically comprehended (Muñoz et al., 2023; Zhang H. et al., 2024). The efficiency of BCIs is in term of computational efficiency (e.g., speed of processing, information transfer rate), ease of operation (e.g., calibration time), energy efficient electrodes, and large-scale implementation have been studied. However, their efficiency in ensuring burden-less enhancements and user safety (Rapeaux and Constandinou, 2021) must also be included. Given this context, the paper presents an ontological framework of BCI interface in mental healthcare.

## Framework for ethics of brain-computer interface in mental healthcare

The state of the research in the domain is largely segmented, siloed, and emphasizes only a few aspects of BCI ethics. BCIs for mental health disorders are exploratory, can have long term psychological effects, and can alter personality, identity, and social relationships (Burwell et al., 2017; Wang et al., 2024; Gruica, 2025; Shaw et al., 2025; Zhang et al., 2025). Affective and psychiatric BCIs modulate decision making and emotions, so that the boundary between a person’s ‘own agency’ and device shaped behaviour is blurred, and often raises ethical concerns of autonomy, consent (Burwell et al., 2017; Steinert and Friedrich, 2020; Sun and Ye, 2023; Livanis et al., 2024) and interaction biases (Hanna et al., 2025). Further, the literature in the domain often emphasises generic ethical considerations in terms of autonomy, consent, dependency on technology (Vlek et al., 2012; Awuah et al., 2024). Such research offers a segmented perspectives about BCI ethics and provides a partial picture.

The need for BCI-specific ethical governance that goes beyond general principles, with targeted measures, long-term monitoring, and multidisciplinary oversight for mental health BCIs (Wang et al., 2024; Sirbu et al., 2025; Wei, 2025; Zhang et al., 2025) has been widely articulated. BCIs are known to alter mood, identity, and develop dependency over time, so the concept of “consent” should be revisited regularly, not treated as a one-off event (Goering et al., 2021; Baker et al., 2023; Shaw et al., 2025; Si et al., 2025). Individuals with psychiatric disorders and cognitive impairments can compromise understanding, appreciation and reasoning about potential BCI risks (Parmigiani et al., 2022; Si et al., 2025). The existing traditional device regulations often focus on safety and efficacy; not on autonomy, mental privacy, and long term support (Wang et al., 2024; Gruica, 2025; Sirbu et al., 2025; Zhang et al., 2025). Multiple studies emphasize the need to revise bioethical principles in the AI/BCI era, and urge to include broader, relational understandings of autonomy, agency, and attention towards stigma, justice, and socio-economic impacts (Glannon, 2014; Sun and Ye, 2023; Livanis et al., 2024; Zhang L. et al., 2024; Shaw et al., 2025).

Evaluation of ethical considerations in BCI research is currently inadequate. Larger concerns exists over obtaining informed consent for BCI interventions due to their unpredictable outcomes, varying expectations, disease related implications and disruptions to daily life (Awuah et al., 2024; Shaw et al., 2025). With rapidly evolving BCIs and high-risk implants, ensuring that patients understand the long-term implications and are continuously updated for consents must be adhered (Awuah et al., 2024; Khan et al., 2024; Satam and Szabolcsi, 2025).

Emerging concepts like neurorights (mental privacy, identity, agency), neural diversity, and BCIs as infrastructures of moral inclusion are add-ons to the existing ethical considerations (Valeriani et al., 2022; Klein et al., 2024; Deshmukh et al., 2025; Gruica, 2025; Wei, 2025). Implantable and commercial BCIs in particular raise substantial safety, privacy, and ethical challenges specifically in terms of risks of unauthorized access and commodification (Boonstra, 2025; Hu et al., 2025; Lavazza et al., 2025; Satam and Szabolcsi, 2025). Risks in terms of device failure and abandonment (e.g., loss of BCI function) can cause serious psychological harm (Gruica, 2025), making maintenance is an ethical obligation.

Implants generate highly sensitive neural data; concerns in terms of unauthorized access, wireless “neuro hacking,” and manipulation of stimulation (El-Osta et al., 2025; Satam and Szabolcsi, 2025; Wei, 2025; Zhang et al., 2025) have to delineated and addressed. Further, the existing data and privacy laws (e.g., General Data Protection Regulation) are not tailored to protect brain data and “mental privacy” (Glannon, 2014; Burwell et al., 2017; Zhang L. et al., 2024; Gruica, 2025). Potential risks in terms of data interception, tampering, and commodification of neural information, fail to address neural privacy (Boonstra, 2025; El-Osta et al., 2025; Satam and Szabolcsi, 2025). The need for robust encryption, secure authentication, tamper resistant design, and strong data protection rules within BCIs (Valeriani et al., 2022; El-Osta et al., 2025; Sirbu et al., 2025; Wei, 2025; Zhang et al., 2025) seems timely critical.

The need for a multi-dimensional framework that encompasses clinical, ethical, legal, sociocultural, and procedural aspects are proposed to be part of BCI specific consent (Si et al., 2025). Conceptual frameworks like the self-implant ambiguity explains of how neuromodulation (e.g., DBS) can alter identity through conceptual, phenomenological, and narrative uncertainties about the self. BCIs may alter self-perception, social identity, and personality, raising worries about changes to personhood and agency (Livanis et al., 2024; Lavazza et al., 2025; Zhang et al., 2025). This may further affect identity, autonomy, and the person’s agency (Shaw et al., 2025). For instance, personality changes and co-agency with devices that impact on selfhood and authenticity are ethical concerns around the use of BCIs (Yuste et al., 2017; Goering et al., 2021; Gordon and Seth, 2024; Shaw et al., 2025). In addition, vulnerable participants (for, e.g., stroke patients with cognitive impairments) raises concerns about coercion, participant suitability, uncertain side-effects, all of which highlights the need for clear criteria and rigorous ethical evaluation in BCI research (Cervera et al., 2018; Awuah et al., 2024; Jia et al., 2025). These concerns has to be effectively and transparently communicated (Vlek et al., 2012).

The need for more ethical policies that protect the autonomy, privacy, and agency of the recipients of care and appropriate measures to uplift clinical effectiveness are needed. For instance, acquiring informed consent from a BCI worn individual is challenging, there are no specified guidelines for BCI researchers to adhere especially while addressing bleeding and infections instances that might occur due to implanted electrodes (Goering and Yuste, 2016; Saha et al., 2021). With multiple experimentations (e.g., brain to brain experiments) and emergence of novel neuro technologies, adequate safeguard mechanisms for private internal realms and individual agency will need to be re-defined and must uphold human rights (Goering and Yuste, 2016). Ethical challenges in EEG-based AI and BCIs pivots around mental privacy, data ownership, informed consent, algorithmic opacity, and intensified by fragmented governance. These factors undermine autonomy, trust, and equity. Further, addressing them requires adaptive governance, explainable AI, dynamic consent, and interdisciplinary collaboration (Mouazen and Bouyed, 2025).

The lawful use of BCIs, along with the protection of privacy and confidentiality is critically important (Saha et al., 2021). The Belmont Report (Department of Health, Education, and Welfare and National Commission for the Protection of Human Subjects of Biomedical and Behavioral Research, 2014), Nuffield framework (Nuffield, 2026), and the *BRAIN Initiative Neuroethics Roadmap* (Eberwine and Kahn, 2020) reflect a progression in neuroethics but each remains limited. Belmont offers strong operational clarity through enforceable principles of consent, beneficence, and justice, yet is outdated for BCI complexities. Further, expansive deployment of AI and big data analytics necessitates a critical re-evaluation of the principles in relation to privacy, confidentiality, data ownership, informed consent, systemic inequities and human dignity (40–42). The Nuffield framework improves contextual relevance by incorporating precaution, autonomy, safety, and equity, but lacks regulatory enforceability. Despite advancements in neuroscience research, innovation, and ethical foresight, the BRAIN initiative is posed with ethical questions in terms of- how can neural activity be modulated while respecting autonomy and identity? How should risks in intracranial studies be defined and communicated, and how can informed consent account for fluctuating decision-making capacity? These issues grow more critical as neural interventions advance (Koroshetz et al., 2018). The BRAIN initiative’s roadmap further expands ethical scope to include agency, identity, and data governance, but remains largely aspirational without actionable standards. Collectively, these frameworks highlight a gap between ethical articulation and implementation, underscoring the need for an integrated, enforceable neuroethics framework for BCI research.

Across several clinical cases (e.g., neurorehabilitation, assistive communication), the lack of an integrated ethical framework has resulted in inconsistent safeguard mechanisms, reinforcing the need for standardized governance, explainability, and user-centric design to ensure responsible deployment (Mouazen and Bouyed, 2025) of BCIs. While, BCIs tend to assure major benefits in communication, rehabilitation, and cognitive enhancement, the existing frameworks are technically fragile and ethically complex. Across recent medical, technical, and philosophical work, three such themes recur. They are: (a) limits on effectiveness, (b) uncertainty about long-term safety and (c) usability, and deep concerns about autonomy, privacy, and justice. More specifically, the general bioethics principles are too abstract, provide little guidance on identity changes and on neuro-rights (Burwell et al., 2017; Sun and Ye, 2023; Livanis et al., 2024; Zhang L. et al., 2024; Shaw et al., 2025). Most of the ethical concerns for BCI are similar to that of medical research (Vlek et al., 2012). The dichotomy between the treatment goals and research goals in BCI often offers unique challenges. Thus, the ethical concerns for specific cases can arise in terms of a conflict between treatment-based interests and research-based interests. For instance, interest towards a BCI intervention can be completely different for a family with an individual with locked in syndrome (LIS), from that of the researchers working towards a development of BCI. This type of mismatch can lead to therapeutic misconception and affects informed decision making and welfare of the individual.

Overall, the landscape of research on BCI ethics offers a fragmented, segmented, and selective approach towards addressing the disconnected parts of BCI ethics, effectiveness, and efficiency. The existing approaches fall short to systemically and systematically address the complex problem of BCI ethics. The ontology of ethics of BCI in mental healthcare is a concise, clear, and comprehensive framework that can fill the gaps in framing the issues.

### Ontology of ethics of brain-computer interface in mental healthcare

**Figure 1:**
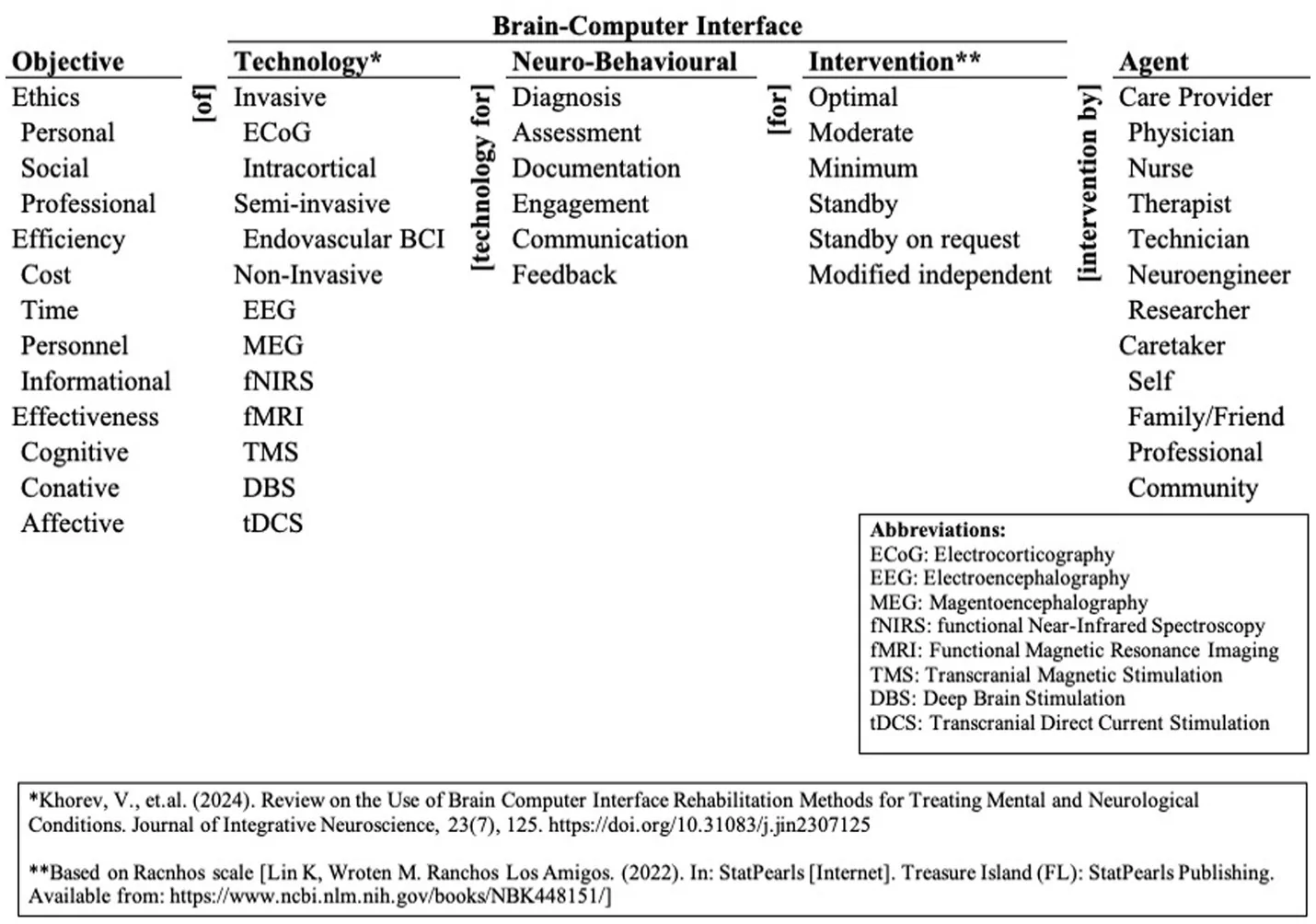
Ontology of ethics of brain-computer interface in mental healthcare.

We use an ontology to describe the challenges, explain the logic, set the expectations, and regulate the outcome of the ethics of brain-computer interface (BCI) in mental healthcare. It is a novel way of framing the challenge. There is no similar framework in the literature to address the issue systemically and systematically. Without a synoptic framework, the present approaches are selective, siloed, and segmented and do not address the ethical challenges holistically as they should be. The “Ontology of Ethics of Brain-Computer Interface in Mental Healthcare” encapsulates the core logic of the challenge clearly, concisely, and comprehensively. The complex interplay of ethics, efficiency, and effectiveness of BCI in mental healthcare can be visualized and analysed using the framework, which is presented in Figure 1 and described below.

### Brain-computer interface (BCI) in mental healthcare

BCIs are being widely deployed in mental healthcare. They are computer-based technologies interfacing with the brain to perform a neuro-behavioural modulation function for a mental healthcare intervention. The three dimensions of BCI (technology, neuro-behavioural, and intervention) are denoted by the middle three columns in the ontology and described next. The BCIs are deployed by agents listed in the last column to fulfil the objectives listed in the first column. We shall discuss them subsequently.

#### Technology of BCI

A BCI technology may be Invasive, Semi-Invasive, or Non-Invasive. The second column (from the left) in Figure 1 denotes the types of technology (a) invasive – in terms of ECoG (Electrocorticography), and Intracortical, (b) semi-invasive- in terms of endovascular BCI, and (c) non-invasive – in terms of EEG, MEG, fNIRS, FMRI, TMS, DBs, and tDCS. An invasive technology penetrates deeply into the brain, a semi-invasive technology penetrates only minimally, and a non-invasive technology does not penetrate the brain or the body of the patient. The sub-types of each of these three types are listed on the second column of the ontology. Each type poses ethical issues that are qualitatively and quantitatively different. They are likely to vary among the sub-types too. The issues related to invasive technologies are likely to be numerous and complex, those related to semi-invasive technologies less so, and those related to non-invasive technologies the least so.

#### Neuro-behavioural stage

The above mentioned technologies are used at neuro-behavioural (third column) and intervention (fourth column) stages of mental health care (refer Figure 1). The three types of technologies may be used clinically for diagnosis, assessment, documentation, engagement, communication, and feedback in mental healthcare. The stages are generally sequential, and a technology may be used in one or more stages. Different technologies may be used at different stages too. The ethical issues related to the use of a technology are the qualitative sum of the issues associated with its stages of use. When multiple technologies are deployed for an intervention, it will be the qualitative sum of the issues for all the technologies for all the stages.

#### Intervention

The mental healthcare intervention using BCI may be at different levels. It may be Optimal, Moderate, Minimum, on Standby as needed, on Standby on request, or Modified Independence, based on the requirement of the patient. These levels are listed in the Intervention column and are in descending order of intensity. The ethical issues at the upper end of the scale are likely to be more numerous and complex, and fewer and less complex at the bottom.

### Agent

The two broad categories of agents that deploy BCI for mental healthcare are the Care Providers and the Caretakers. They are listed in the last column of the ontology. The care providers may be physicians, nurses, therapists, technicians, neuroengineers, or researchers. The caretakers may be oneself, family/friend, professional, or the community. These are listed as subtypes of agents in the last column. The ethical requirements of the agents will be determined by the BCIs (Technology + Clinical Stage + Intervention) they must deploy.

### Objective

The objective of BCI intervention for mental healthcare by an agent is to balance the efficiency of the intervention, its effectiveness, and its ethics. These are listed in the first column of the ontology. The desired effectiveness may be cognitive, conative, or affective; the efficiency may be in cost, time, personnel, or informational; and the ethics may be personal, social, or professional. Effectiveness is the primary threshold for the use of BCI – it is the requirement that the technology must have the intended and desired effects. The technology must also be efficient in its resource use for it to be accepted and used. A very costly technology, one that is time-intensive, personnel-intensive, and information-intensive will face significant barriers to acceptance and use. Effectiveness is necessary but not sufficient; it must be balanced with efficiency. Last, the performance must be balanced against the ethics of using the technology. The ethics can be a showstopper. An unethical technology would be unacceptable despite its effectiveness and efficiency.

The primary motivations for the adoption of BCI are the gains in the efficiency and effectiveness of mental healthcare by the agent. There is likely to be a trade-off between efficiency and effectiveness, and between the attributes of efficiency and effectiveness. For example, a cognitively effective technique may require a long time and consequently costly. The cognitive effectiveness must be balanced against temporal and cost efficiency. If the technique requires a highly paid care provider to be effective, the cost efficiency may decrease further. The increased time efficiency of a highly paid care provider must be balanced against the lower potential cost efficiency of the same. In choosing the trade-off between the efficiency and effectiveness of the technique, the agent must weigh the personal, social, and professional ethics of the decision. The professional guidelines may limit the choice of the agent. The choice may also be driven by its social acceptability and the personal preference of the agent based on prior experience. These are complex trade-offs in a newly emerging field that does not have well developed guidelines. The framework allows the agent or his/her surrogate to formulate the problem, analyse the issues and design a solution.

In sum, the ontology encapsulates 9 × 10 × 6 × 6 × 10 = 32,400 pathways for ethics of brain-computer interface in mental healthcare. Of these, three illustrative pathways are:

1. Personal ethics of invasive ECoG technology for diagnosis for optimal intervention by care provider physician.
2. Cost efficiency semi-invasive endovascular BCI technology for documentation for minimum intervention by care provider therapist.
3. Affective effectiveness of tDCS technology for feedback for modified independent intervention by caretaker community.

Together, the ethics, efficiency, and effectiveness outline the overall ethics of brain-computer interface in mental healthcare: in terms of its technology, neuro-behavioural intervention and the agent of care. The application and development of the ontology follows the description of the logic and process as put forth by Ramaprasad and Syn (2014, 2015). The ontological method is used to study ethics of neurocognitive rehabilitation (Chandra et al., 2026), mental healthcare (Martínez et al., 2022; Chandra et al., 2024), COVID-19 strategies (Sreeganga et al., 2021), public health informatics research (Ramaprasad et al., 2017), and mHealth research (Cameron et al., 2017).

The following section describes how the ontology can be used to articulate the ethical challenges of BCI in mental healthcare.

## Ethical challenges of BCI in mental healthcare

The ethical challenge of BCI in mental healthcare of a patient by an agent can be defined using the ontology to:

- Specify the BCI requirements of a patient in terms of:
  - The BCI technology.
  - The clinical steps.
  - The recommended interventions.
- Specify the agent(s) for the interventions.
- Specify the ethical requirements of the BCI interventions, and the trade-off between the ethics, efficiency, and effectiveness of the interventions.

Invasive, non-invasive, and external BCIs pose different sets of ethical issues that are distinctive and overlapping. The following discussion is organized around the three types. Within each section we discuss the ethical issues for care providers, caretakers, and digital agents.

The triangulation of how ethics, effectiveness, and efficiency interact, overlap and constrain each other in the development and implementation of BCIs is the core of the problem. The problem must be studied synoptically and not in siloes. The following section discusses the 3 Es in detail. The 3 Es are robust; the synergy between them must be delineated, and the trade-offs should be used to improve BCIs that are socially responsible, safe, and aligned with human values. Presently, the 3Es are segmentally defined and understood; the need for a comprehensive understanding is critical for the domain.

### Ethics

Medical and non-medical applications of BCIs and its associated ethical concerns has to be defined distinctively (Davidoff, 2020; Zhang H. et al., 2024), especially in terms of user-centric and societal safety-centric (non-maleficence) ethical concerns. In addition, ethical concerns over the three systems used in BCIs in terms of the sensor, the decoder, and the translator (Davidoff, 2020) must be systemically defined to ensure sense of agency and accountability. Despite ease of access, ethical concerns over non-invasive eBCIs in terms of erosion of privacy, mental monoculture, loss/gain of autonomy, inequality, and cheapened achievements are widely articulated (Gordon and Seth, 2024).

Ethical issues in BCIs have raised legislative and societal concerns. Legal concerns in terms of testimony, consent and capacity (e.g., consent for medical treatment by speech-impaired individuals), and harm have to be critically examined and dealt with while using BCIs for neural-decoded speech (Chandler et al., 2022). Neuro-security, for instance, poses similar ethical concerns over brain data and mental privacy (Ruiz-Vanoye et al., 2024). While brain data security focuses narrowly on protecting neural information, neuro-security addresses holistic protection of the brain as a whole. Neural privacy in terms of access and use of real-time brain data, potential data breaches, and surveillance of varied recipients of care must be regulated.

The need for an objective medical framework for BCI to understand the possible infringement of neural privacy and an individual’s sense of personal identity (Muñoz et al., 2023; Zhang H. et al., 2024). Neuro-rights in terms of free will, cognitive liberty, brain data security, and mental privacy are critical legislative components to protect personal autonomy and freedom of thought of the individuals (Muñoz et al., 2023; Ruiz-Vanoye et al., 2024; Shaw et al., 2025).

Ethical concerns over deep-brain stimulation, especially in terms of potential risks and side effects, the influence of such an implant on the self-concept, its effect over autonomous decision making, post-treatment autonomy, and unintended consequences are widely articulated in the literature (Clausen, 2013; Shaw et al., 2025). Despite the cutting-edge research, concerns of ethical governance (Zhang J. et al., 2024), sense of agency, autonomy, neuro-data privacy across the levels of interface (e.g., cortical versus neural) and the type of BCI modalities have to be defined and integrated. BCIs enhance autonomy by restoring communication and control in otherwise “locked-in” or severely disabled patients, improving perceived independence and quality of life (Awuah et al., 2024; Khorev et al., 2024; Magalhães et al., 2025). However, closed -loop devices risk blurring the boundary between the user intention and device-driven action, raising concerns over the “true agent” of behaviour and how responsibility is attributed if impulse control or moral judgement are altered (Hramov et al., 2021; Awuah et al., 2024; Zhang J. et al., 2024).

Similarly, BCI interfaces intended to optimize cognitive or emotional preferences raise concerns about enhancement, fairness, and the social pressure to adopt such technologies in competitive or resource-limited contexts (Hramov et al., 2021; Saha et al., 2021; Awuah et al., 2024). These and other similar challenges pertaining to neuro-data privacy, neuro-rights, informed consent, and clinical governance have strengthened the neuro-data protection regimes to arrive at global ethical guidelines to protect privacy, prevent bias, and ensure equitable access (Saha et al., 2021; Angulo Medina et al., 2024; Awuah et al., 2024; Liu J. et al., 2025).

### Effectiveness

BCIs effectiveness in terms of symptom relief and assessment is well-documented; nonetheless, their effectiveness for specific conditions is yet to establish clinical benchmarks for diagnosis and therapy. The need for culturally attuned generative AI to integrate contextual norms to ensure equitable and inclusive decision making in mental healthcare is critical (Zaboski and Muller, 2026). Concerns over the compatibility of sensors and electrodes with human brain tissues must be addressed to enhance the effectiveness of the BCI application (Zhang J. et al., 2024). Techniques like emotional modelling have effective system designs that define, distinguish and classify signals. Advancements in machine learning and adaptive algorithms are potential pathways to provide individualised and ethically aware affective BCIs (Xiong, 2025).

Efforts towards an integrated understanding of BCIs effectiveness must be formulated to further inform research and practice on dimensions of ethical and efficient use of BCI technologies. Methods for evaluating the effectiveness of BCI-assisted diagnosis or treatment for different diseases and functional impairments still need to be established. In addition, clinical trials and ethical standards for new medical devices are also under construction (Burwell et al., 2017; Ma et al., 2025).

Evidence suggests the effectiveness of BCI in terms of symptom control, communication, and functional recovery in psychiatric and affective spectrum conditions. DBS, for instance, has demonstrated effectiveness in treating Treatment Resistance Depression (TRD) with selective and targeted stimulation of specific brain regions (Hong, 2024). BCI’s effectiveness in terms of diagnosis, assessment and feedback for mild depressive disorder (MDD) has provided profound insights into the clinical and neural mechanisms of MDD (Elnaggar et al., 2025).

Despite the integration of artificial intelligence in DBS, challenges in terms of precision medicine, diagnostic models, and patient classification still restrict the clinical benefits that explainable AI could potentially deliver (Allen, 2023). BCIs and Neuro-prostheses are futuristic solutions for neurological conditions, the need for supervision by governmental and nongovernmental organizations to define technical standards, medical and manufacturing guidelines, and monitoring the long-term effects (Mikołajewska and Mikołajewski, 2013) are needed to facilitate integration of technologies for neurorehabilitation. Future research towards addressing the challenges like device portability, clinical validation, signal accuracy (especially in terms of decoding) and multimodal integration could strengthen BCI’s transformative potential and personalized rehabilitation protocols (Ma et al., 2025).

### Efficiency

BCI technologies provides evaluation and functional status alongside it’s neural rehabilitation training and regulation. For instance, BCI combined treatments have improved upper limb motor function, ensured good safety and quality of life amongst stroke patients (Liu Q. et al., 2025). It can determine the next diagnosis, treatment, and rehabilitation training plan based on its ability to use BCI and further combine with other intervention methods to help functional recovery (Chai et al., 2024). Further, therapeutic AI systems used for mental health conditions should be engineered to resist uncritical validation of patient inputs while ensuring that their decision-making processes remain transparent, interpretable, and fully auditable within a clinical governance framework (Zaboski and Muller, 2026).

Long term efficacy and benefits of BCI needs to be analysed and evaluated. Assurance of progress monitoring and full life cycle technology support for mental health conditions are highly critical while nation states are implementing emerging neurotechnology (UNESCO, 2026). Feedback from subjects with DBS device implants affirms the need for end-user input to address concerns like the need for control over device function (e.g., conscious intentional control versus computer algorithms), and the user consent (e.g., individual user autonomy, consent to therapy) (Klein and Nam, 2016).

The legislative need to distinguish between general privacy and mental privacy in the context of neurotechnology is critical (Brown, 2024). For instance, neural data is particularly sensitive and private. The recent advancements in implantable brain machine interface have optimized improvements in the neural interface technology (e.g., enhancements in information transfer rate, and calibration strategies) and have enhanced scalability of new surgical workflows (Rapeaux and Constandinou, 2021), which is a remarkable scientific milestone.

### Balancing ethics, effectiveness, and efficiency

The ethical regulation of a newly emerging technology evolves more slowly than the establishment of their effectiveness and efficiency. The determination of clinical efficiency and effectiveness of BCI dominantly fall within the domain of care providers – the physicians, nurses, therapists, technicians, neuroengineers, and researchers. Delineating the ethics of BCI must include the caretakers – the self, families/friends, professionals, and the community. The care providers are likely to have a direct knowledge and understanding of the technology and its application. They have greater agency in determining the effectiveness and efficiency of BCI. However, the caretakers are likely to have direct experience and understanding of the recipients and effects of the technology. They have greater agency in determining the autonomy, privacy, justice, and equity of use of the technology. Thus, the priority of the two groups of agents and their subgroups with reference to efficiency, effectiveness, and ethics are likely to be different because their roles and stakes in BCI are different. Given the differences in their knowledge, stake, and the outcomes sought by the agents, it is a complex challenge to balance their perception of the safety, justice, and equity of using the technology. Despite the complexity of the challenge, any loss of confidence in the ethics of the technology may severely (if not fatally) affect the use of the technology for a long time. Such confidence will be critical to promoting urgency to adopt it rather than hesitancy to do so. The ontology unravels the large number of BCI pathways connecting the objectives to the agents via the combination of technology, neuro-behavioural intervention stage, and the level of intervention. The following is some of the pathways and the associated ethical issues.

Development of BCIs for mental health requires a careful balance between ethics, efficiency, and effectiveness. The balance is critical especially given the stakes of safety of the participant (e.g., vulnerable patients) and the access to intimate data (e.g., neurodata, neuroethics). BCIs must be efficient and usable in real world settings, current BCIs are challenged in terms of technical inefficiencies, steep learning curves, demands training, emotional burden, psychological harm, and interaction biases (Glannon, 2014; Burwell et al., 2017; Coin et al., 2020; Angulo Medina et al., 2024; Hanna et al., 2025). For instance, ECoG strips are efficient invasive BCIs which assists people with epilepsy or Amyotrophic Lateral Sclerosis (ALS) to control devices like robotic limbs or to communicate (Rapeaux and Constandinou, 2021). Studies using ECoG have demonstrated effective real world applications, long term stability and have been efficient by reducing cost and complexity. However, in such scenarios, prioritizing efficiency and clinical effectiveness without strong ethical safeguards can lead to violations of privacy, autonomy, mental integrity, cultural insensitivity, unequal access, and dependency on immature technologies (Wang et al., 2024; Zhang Z. et al., 2024; Deshmukh et al., 2025; Sirbu et al., 2025; Wei, 2025).

BCIs vary in terms of their level of invasiveness and functions (read vs. write). These differences create the trade-offs between performance (effectiveness and efficiency) and ethics. The need to formulate guidelines on parameters of risk level, function, autonomy, equity and social impact are critical. As discussed earlier, several ethical frameworks are being applied to guide BCI use (Coin et al., 2020; Livanis et al., 2024; Wang et al., 2024; Zhang Z. et al., 2024; Wei, 2025). In absence of integrated framework, precise governance and stringent security thresholds towards implementation of distinct regulations that safe guard read-out versus write-in BCIs needs to be deployed (Coin et al., 2020; Sun et al., 2023; Livanis et al., 2024; Wang et al., 2024; Wei, 2025).

The domain of BCI has multitude of issues concerning ethics, effectiveness, and efficiency. The discussions amongst researchers, ethicists, and public are fragmented and focuses on different issues (Nijboer, 2015). Available BCI technologies balance ethics, effectiveness, and efficiency in several possible ways rather than one best solution. Invasive BCIs despite being more powerful, they raise deeper ethical and regulatory concerns. Non-invasive BCIs emphasize safety and scalability, but face performance limits.

Ethical governance frameworks for BCIs emphasize rigorous informed consent procedures -particularly for cognitive vulnerable populations -along with robust protections for neural data, equitable access, and continuous long-term monitoring. Further, mandates on transparency about algorithms and limits of benefit, reinforced by interdisciplinary collaboration across clinical, technical, and regulatory domains are widely articulated (Wang et al., 2024; Zhang Z. et al., 2024; Sirbu et al., 2025; Wei, 2025).

While classical cases of ethical violations in BCI-based mental healthcare remain limited due to the field’s nascency, a growing body of literature identifies critical risk domains—including neuroprivacy breaches, autonomy erosion, device abandonment, disability justice and informed consent deficiencies that function as early indicators of potential systemic ethical failures (Peabody Smith et al., 2025; UNESCO, 2025; Zhang et al., 2025). Integrating readiness assessment methodologies (RAM), alongside ex ante and ex post evaluation frameworks, within robust regulatory and governance structures for neurotechnology is critical to ensuring equity, justice, data protection, and lifecycle support, thereby enhancing the overall efficiency and effectiveness of BCI implementation (UNESCO, 2026).

## Conclusion

The challenges for BCI technologies are broadly two-fold. Firstly, there are technical challenges and constraints; and secondly, the challenges in terms of integration of BCI technologies that equally address the 3Es.

The need for mechanisms to understand and optimize biomarker triggered network (temporally), stimulation site selection, and therapeutic responses (Clausen, 2013; Oganesian and Shanechi, 2024; Sellers et al., 2024) is critical for building a robust closed-loop BCI technology for neuropsychiatric disorders. A comprehensive understanding of technical challenges in terms of scalability and portability, electrode stability and yield, and information transfer rate must be arrived at to resolve the complex challenge faced by intracortical BMIs (Rapeaux and Constandinou, 2021).

Challenges in terms of technical constraints (e.g., prevention of inflammation and other damages due to BCI implants); complexity of the neural circuitry underlying the perceptual, cognitive, and motor processes; and the brain’s plug-and-play mechanisms (Gordon and Seth, 2024) further concern the efficiency and effectiveness of the BCIs. BCIs systems must advance their implant biocompatibility, neural- recording capability, while incorporating a well calibrated refinement techniques that support device adaptation, closed- loop learning, and reduce surgical risk (Davidoff, 2020). Data-driven machine learning and system design approaches are relevant to provide futuristic BCI pathways (Oganesian and Shanechi, 2024).

Inadequate research and lack of recommendations in the literature for handling concrete ethical concerns (Burwell et al., 2017) have been a foundational issue in the domain. There is a larger need for interdisciplinary collaboration to address the complex ethical and legal issues in the domain of BCI. For instance, the concept of autonomy remains overarching, poorly defined, and debated; despite the neuromodulation techniques like BCI and DBS have been successful in restoring autonomy and agency for patients (Glannon, 2014; Burwell et al., 2017). Further, the constant need for enhancing BCI’s utility and access continues to challenge the researchers in the domain (Chandler et al., 2022).

The above challenges reinforce the importance of scrutinizing BCI technologies across the triad of ethics, effectiveness, and efficiency to deliver measurable benefits without compromising on patient safety, privacy, agency and autonomy. Lastly, the BCI’s and emerging technologies should address the larger questions of scientific pluralism, segway from reductionist approach to that of integrated, systemic and systematic understanding of mental healthcare. Towards this end, the framework (Figure 1) presents a cognitive map to integrate ethics, effectiveness, and efficiency as the impetus for developing the upcoming BCIs, revisit and modify the existing BCIs, and innovate from the learning and feedback in the paradigm of neuro-behavioural modulation of mental healthcare.

## Data Availability

The original contributions presented in the study are included in the article/supplementary material, further inquiries can be directed to the corresponding author.
